# Supplementary material for: Characterization Studies on the sugC Gene of Streptococcus suis Serotype 2 in Adhesion, Invasion, and Virulence in Mice
Source: Vet Sci. 2024 Sep 21;11(9):447. doi: 10.3390/vetsci11090447 (PMC11435659; doi:10.3390/vetsci11090447)
Supplement: Supplementary file 1 [file vetsci-11-00447-s001.zip › Supplementary Material Figures-updated.pdf]

Supplementary Materials:

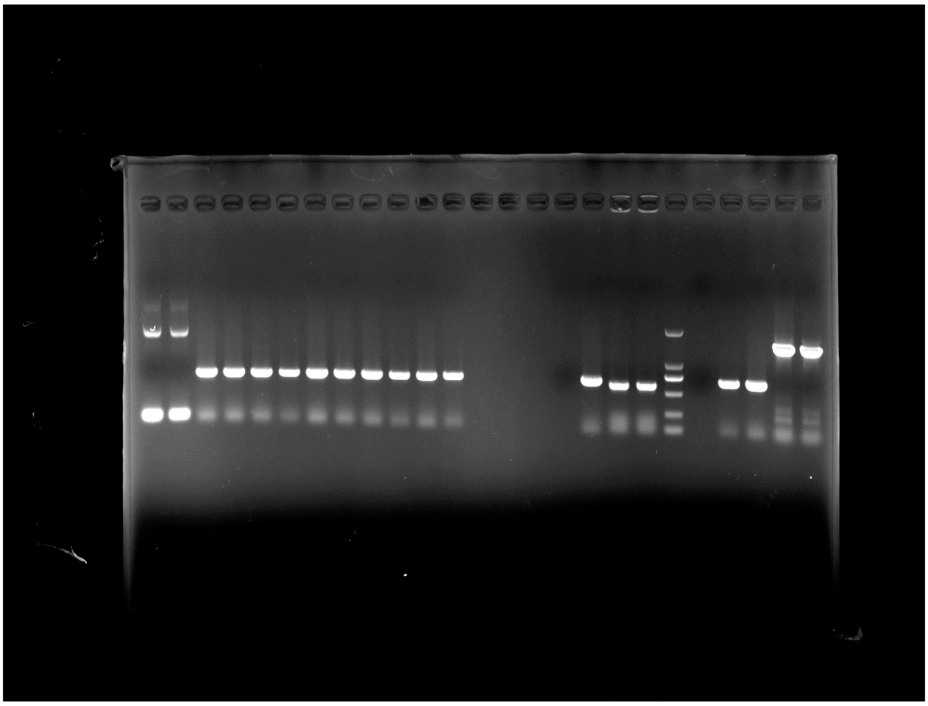

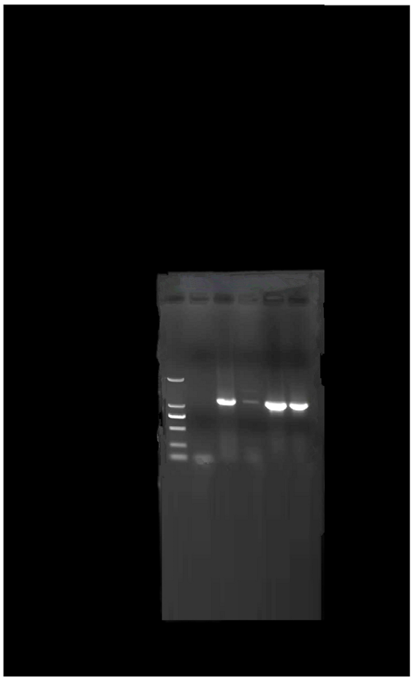

Figure S1. The original gel images of Figure 1.

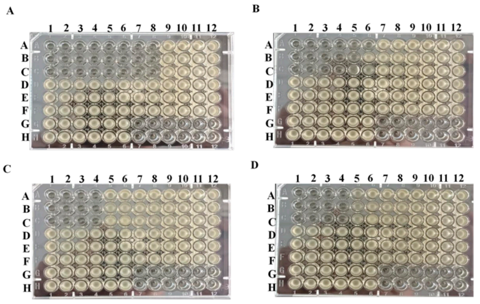

Figure S2. The results of MICs for Δ*sugC*, CΔ*sugC*, and TJS75. The effectiveness test results of GEN to *E. coli* ATCC 25922. Line1-line12: 128 μg/mL, 64 μg/mL, 32 μg/mL, 16 μg/mL, 8 μg/mL, 4 μg/mL, 2 μg/mL, 1 μg/mL, 0.5 μg/mL, 0.25 μg/mL, 0.125 μg/mL, 0.0625 μg/mL. (B), (C)and (D) were the MICs for Δ*sugC*, CΔ*sugC*, and TJS75. Line1-line12 of (B), (C) and (D) were similar to (A).
